# Characteristics, challenges and innovations of waste picker organizations: A comparative perspective between Latin American and East African countries

**DOI:** 10.1371/journal.pone.0265889

**Published:** 2022-07-29

**Authors:** Jaan-Henrik Kain, Patrik Zapata, Adalberto Mantovani Martiniano de Azevedo, Sebastián Carenzo, Goodluck Charles, Jutta Gutberlet, Michael Oloko, Jessica Pérez Reynosa, María José Zapata Campos

**Affiliations:** 1 Gothenburg Research Institute GRI, University of Gothenburg, Gothenburg, Sweden; 2 School of Public Administration, University of Gothenburg, Gothenburg, Sweden; 3 Centro de Engenharia, Modelagem e Ciências Sociais Aplicadas, Universidade Federal do ABC, Santo André –SP, Brazil; 4 CONICET and Instituto de Estudios sobre la Ciencia y la Tecnología, Universidad Nacional de Quilmes, Bernal, Buenos Aires, Argentina; 5 University of Dar es Salaam Business School, Dar es Salaam, Tanzania; 6 Department of Geography, University of Victoria, Victoria, BC, Canada; 7 School of Engineering and Technology, Jaramogi Oginga Odinga University of Science and Technology, Bondo, Kenya; 8 Instituto Interdisciplinario de Ciencias Sociales, Universidad Centroamerica (UCA), Managua, Nicaragua; 9 School Business, Economics and Law, University of Gothenburg, Gothenburg, Sweden; Cranfield University, UNITED KINGDOM

## Abstract

Waste picker organisations (WPOs) around the globe collect, transport and process waste to earn their living but represent a widely excluded, marginalised and impoverished segment of society. WPOs are highly innovative, created by grassroots out of “nothing” to deliver economic, social and environmental sustainability. Still, we do not know how such innovations are developed, and how they are disseminated and adopted by other groups. This article examines characteristics, challenges and innovations of WPOs across five countries in Latin America and East Africa. It is based on quantitative and qualitative data regarding modes of organisation and management, gender, received support, business orientations, environmental and social contributions, and innovations developed in response to multiple challenges. The paper provides a comprehensive understanding of WPOs’ activities and their grassroots innovations in the Global South. The study shows how WPOs contribute significantly to the economic, social and environmental sustainability of the societies they serve as well as the wider urban societies. To start and maintain WPOs in informal settlements with a lack of infrastructure, institutional frameworks, and public and private investors is a difficult quest. WPOs take many different organisational forms depending on the complexity of local realities, ranging from advanced collective organization as cooperatives to small self-help groups and microentrepreneurs. Self-organisation into regional and national networks provides economic opportunities, autonomy and stability as well as political influence. Yet, institutional support is fundamental and the lack thereof threatens their existence. Sustaining WPOs as important providers of socio-environmental benefits through governmental and non-governmental actions is a worthwhile undertaking that builds sustainability.

## Introduction

### The world of informal waste pickers

Waste pickers around the globe collect, transport and process waste to earn their living. When doing so, they make a significant contribution to reducing the carbon footprint of cities [1–3], recovering resources, improving environmental conditions and health of low-income residents, and creating jobs and income among the poor. They are also active in waste management policy making, thus decentralizing and democratizing policy processes [4, 5]. Yet, they represent one of the most widely excluded, marginalised and impoverished segments of society [6].

Waste pickers are exposed to contaminated materials [7]; suffer from widespread prejudice and stigmatization [8, 9]; are persecuted by police as waste picking is typically illegal [10]; experience difficulties to create formal cooperatives or associations, including access to funding [11]; usually depend on exploitative relations with intermediaries and are susceptible to global market oscillations [12]. All these structural difficulties lead to persistent poverty and hampers the contribution provided by this sector to a Circular Economy [13].

Waste pickers organize themselves in different ways, such as youth groups, women groups, extended family groups, community-based organizations (CBOs), cooperatives, associations, networks and micro-enterprises [12, 14]. These waste picker organisations (WPOs) are fundamental as platforms for improving working conditions, enhancing quality of life (by raising self-esteem, health, etc.), advocating for political power and increasing responsiveness to their demands from governments and other actors [15]. WPOs might receive different forms of financial or infrastructure support and technical advice at start-up [16], many continue to depend on governmental or non-governmental organization (NGO) support to access operating space, machinery, equipment and professional training, or on advertising campaigns to promote source separation of recyclables in households [10, 12].

Maintaining autonomy and self-governance is a challenge, particularly for groups with initial governmental support or benefitting from early public-private partnerships [12]. Yet, waste pickers have managed to build second grade organizations (e.g. networks, federations or trade unions) playing key roles in backing demands to be recognized and formalized as recycling service providers [4, 17, 18].

Access to space, infrastructure (water, sanitation, electricity) and amenities is vital for WPOs to operate efficiently and safely. It is also important to have officially approved and controlled waste transfer points where collected waste and recyclables are stored [19, 20]. Good relationships with local governments are thus vital but municipalities often remain hesitant towards WPOs [15]. Also, support of WPOs clashes with the interests of powerful corporate waste management actors and other players of the private sector. Most countries still lack national regulatory frameworks recognizing WPOs as waste management actors [21]. For example, in view of increasing climate change related urban flooding, the collection and recovery of recyclable materials from household waste by WPOs helps reduce water-logging [22] but this contribution largely remains unrecognised. All in all, local authorities tend to be reluctant to acknowledge WPOs’ role in the waste system and do not fully support waste picker initiatives. WPOs habitually experience discontinuities or unfulfillment of agreements and contracts established with local governments [10, 23–25].

In spite of the valuable environmental, social and economic contributions provided by WPOs, these are still under-researched and existing research is typically qualitative and case based. Although such studies provide context-specific insights, there are knowledge gaps in understanding how WPOs can thrive and become resilient. Recent qualitative research has revealed the knowledge, competences, innovative capacities and services developed by WPOs when designing new recycling processes and infrastructures, even though they often do not follow scientific procedures and technological standards [26]. Yet, we do not know much about how innovative solutions are developed by WPOs, and how they are disseminated or adopted by different groups. According to Hardoy et al. [27] this usually entails a lengthy process, but what are the hurdles and what are the drivers to promote the dissemination of grassroots innovation?

Informed by the grassroots innovation literature, this article examines the characteristics, challenges and innovations of WPOs, including specific gender aspects, in a comparative study in five countries in Latin America and East Africa. It is based on quantitative and qualitative data regarding the modes of organisation and management, gender aspects, received support and advice, business orientations, environmental and social contributions, and innovations developed in response to the multiple challenges. By doing so, this article contributes with an overview perspective and a more comprehensive understanding of WPOs’ contributions and specifically of women in this sector to the social and environmental sustainability of waste management systems in the Global South.

### Grassroots innovation

Approaches critical to the mainstream Schumpeterian innovation model have extensively shown its shortcomings when it comes to addressing the needs of poor and vulnerable social groups [28] and have found that such innovation may even reinforce existing social and economic inequalities [29]. Grassroots innovation movements are, therefore, receiving increased attention from scholars interested in issues of environmental governance driven from below [30, 31]. The grassroots and inclusive innovation literature has evidenced the key role of social movements and CBOs in generating novel bottom-up solutions to address local needs, interests and values [30–32]. According to Gupta [33], such initiatives can contribute significantly to reducing poverty, increasing social inclusion, creating gender equity and other objectives covered under the Sustainable Development Goals (SDGs).

Nevertheless, grassroots and inclusive innovation cannot be studied and understood in relation to single grassroot actors or a specific culture. Such innovations usually develop through complex networks of civil society organizations, practitioners, activists and other grassroots, generating bottom-up and novel solutions that involve the resources, knowledge, interests and values of local communities [29, 34]. The bricolage literature, for example, has explored how social entrepreneurs overcome scarcity by ‘making do with what is at hand’ [35, 36]. Grassroots innovations and initiatives are hence organized differently and develop different characteristics according to the diverse challenges they face. They emphasize ingenuity that fits the needs of their communities and primarily concern local change and empowerment of local communities, but may also raise awareness about structural obstacles to prompt change in mainstream institutions towards systemic transformation [29]. This implies that studying grassroots innovation requires a relational perspective, attentive of power relations and social asymmetries. Heeks, Foster and Nugroho [37] have stressed the tensions between heterogeneous but converging bodies of practice and knowledge. Following this vein, Vasconcellos, Dias and Fraga [38], have taken into account the incidence of visible and invisible gender biases that may dismiss the role of women within the innovation processes.

Bottom-up entrepreneurial practices, shaped by the lack of resources, have the potential to affect failing conventional systems in times of crises and emergency situations [39]. But grassroots innovations operate in “niches” outside of mainstream systems [40], where social inequalities [41] and epistemic asymmetries [42] influence how these innovations are acknowledged (or not) by more formalized or institutional actors. When analysing innovation and inclusion in the Global South, we therefore also follow Fressoli, Dias and Thomas [43] regarding the importance of situated approaches, rather than assuming universality of grassroots innovations beyond local and regional specificity. Recent contributions have further underlined the relevance of such a perspective when analysing waste pickers´ innovations in Africa and Latin America [44–47], e.g. considering institutional settings and policymaking oriented towards a local implementation of a circular economy of waste.

## Methods

The article is informed by a quantitative survey of WPOs and a qualitative follow-up through in-depth interviews with members of the same WPOs. The study is part of an ongoing research program on WPOs developed over the past decade by research team members on informal waste management in the Global South. It was implemented in contexts where researchers were already embedded in the local contexts and had access to key actors, often in the form of advocacy research [48]. The studied countries are Argentina, Brazil, Kenya, Nicaragua and Tanzania. Argentina and Brazil in South America have a longer history in collective organization of waste pickers, while Nicaragua exemplifies more emergent and recent recycling movements in Central America. Kenya and Tanzania represent less articulated WPOs in East Africa, where waste picking is a resilient activity for large populations living in extreme poverty. The focus within each country was on the respective metropolitan areas of Buenos Aires, São Paulo, Managua, Kisumu and Dar es Salaam.

Due to a long-term engagement with informal waste management in these countries, the research team could conduct a broad data collection, involving informal waste pickers and other key actors well known to us in helping identify more recent initiatives through snowball sampling. The selection criteria for participating in this study was that the WPO had constituted a group that was operative at the moment of the survey, but did not necessarily have to be legally registered. Framing of the collected data was supported by previous long-term ethnographic and participatory action-research conducted by the involved researchers in the five countries.

Our research follows the research ethics protocol of the involved universities, in terms of guaranteeing consent, confidentiality and privacy, as well as minimizing risks and possible harm as a consequence of the research. For the Swedish project, based on national guidelines and previous decisions by the Central Ethical Review Board for similar projects, it was deemed that no ethical review was needed since no sensitive personal data were collected. Additionally, all data have been managed according to GDPR guidelines. The Canadian project received ethical approvement (reference number 17–193). Full informed consent by respondents was always obtained (oral or written depending on situation and context).

The survey was divided into four main sections with 28 questions in total: background; collection, processing and commercialization; employment; and relationships [see S1 Appendix]. The surveys were carried out by local researchers in the five countries visiting each of the waste initiatives. All in all, 123 WPOs were involved in the survey: 16 in Argentina, 21 in Brazil, 48 in Kenya, 10 in Nicaragua, and 28 in Tanzania. In Kenya and Tanzania, WPOs are often smaller in size and thus spread over a larger number of WPOs.

The survey data was complemented by 45 in-depth interviews with the same groups, leading to the re-categorization of some WPOs, e.g. “other type of initiatives” was subdivided into “cooperative”, “CBO” or “private company”. This study applies Civil Society Organizations (CSOs) as a wider umbrella term for “all non-market and non-state organizations outside of the family in which people organize themselves to pursue shared interests in the public domain” [49] but it should be noted that, in relation to the data from Kenya and Tanzania, a strict categorization into “for profit” or “not for profit” initiatives was difficult to apply, since many of the “not registered” initiatives plan to operate as family businesses or CBOs. Also, both registered and not registered businesses often have ambitions to simultaneously serve the community as social entrepreneurs. The same most often also applies to the Latin American cooperatives, where members recognize their position as service providers to the local community while also adding value and commercializing the collected recyclables.

The criteria for selecting WPOs for in-depth interviews included evidence of longer operations and development of significant innovations. These semi-structured in-depth interviews were carried out by local researchers [26], following a common interview guide [see S2 Appendix] The informant was usually a representative of the management of the WPO and an interview took 1–2 hours and was conducted at their workplace. Each interview was audio-recorded and partially transcribed for analysis. We asked the participants to share the origin of their initiative, difficulties they had experienced since the start and how they addressed these difficulties. We further wanted to know what they felt were their key achievements and what kind of innovations they had developed. We especially probed into opportunities they have had in the past and what they did to take advantage of these opportunities since, sometimes, important achievements and innovations are not always obvious to the WPOs themselves. Finally, we wanted to know about their future plans and ambitions. Both surveys and interviews were carried out in the most suitable local language, e.g. Dholuo, English, Kiswahili, Portuguese or Spanish.

We conducted thematic content analysis [50, 51] of the interview data, following the themes from the interview guide but with ambition to uncover new categories and issues emerging from the analysis, where some examples included issues such as resource mobilization, identity formation and opportunities for market expansion or creation.

## Results

### Organization, membership and governance

**Fig 1:**
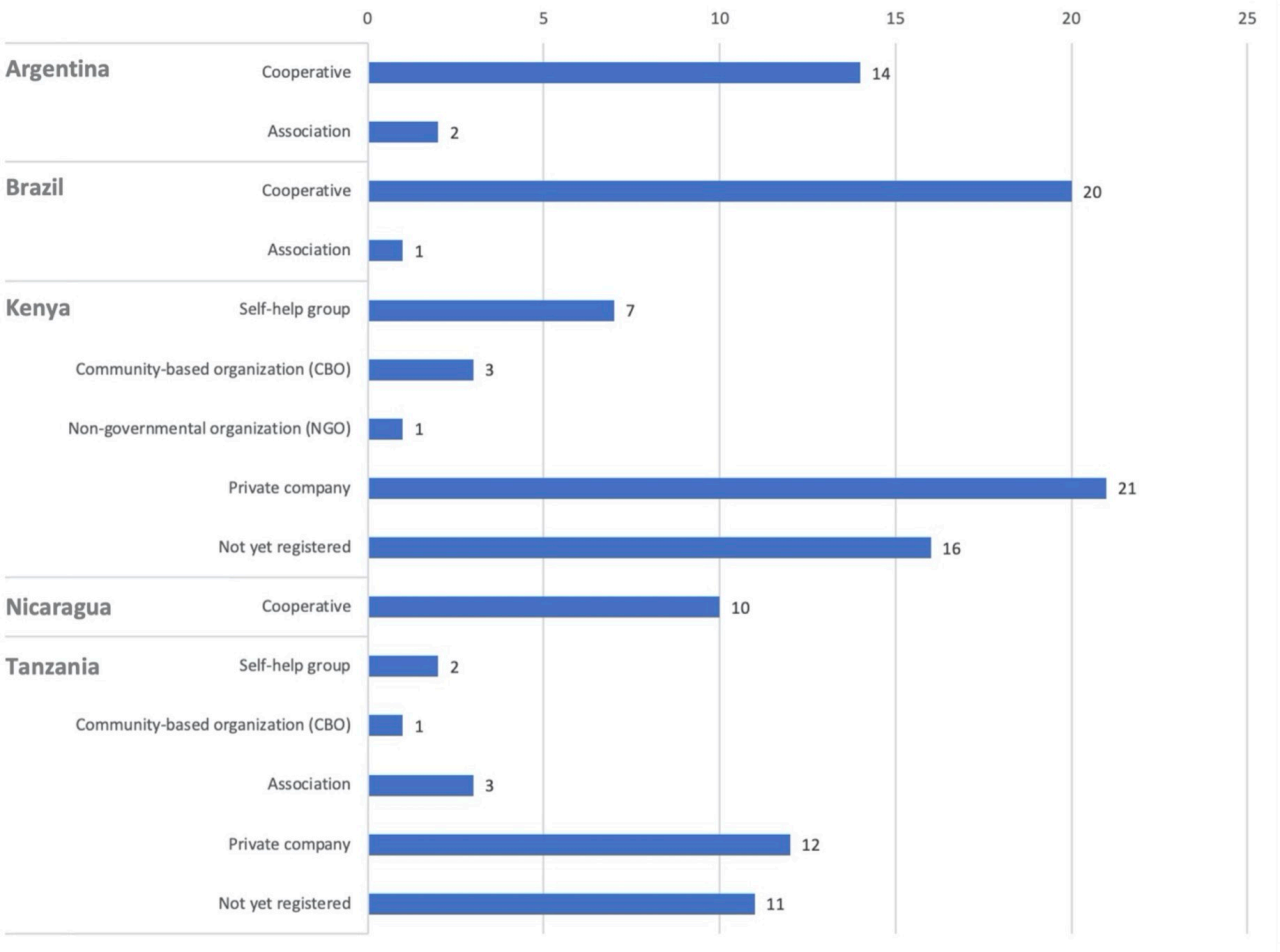
Types of waste picker organizations across the different countries.

Across all five countries, two types of organizations are dominant: cooperatives and small-scale private companies. There are also many initiatives that are not yet formally registered. Breaking it down per country, there is a clear difference between Latin America and East Africa (Fig 1). In Argentina, Brazil and Nicaragua, the cooperative is the prevailing form of collective organization, explained by the historical development of the social and solidarity economy movements in this region and by the long-term policy promotion of cooperatives [52]. Studied associations are also operating in a format similar to the cooperatives.

In Kenya and Tanzania, self-help groups and CBOs are frequent. These can be seen as a parallel to the Latin American cooperatives, but in less advanced stages of organizational development, as we elaborate on below. In our African context, the private company is a dominant form of organization, with some of the companies in Tanzanian being fairly large. The interviews indicate that many of the “not yet registered” organizations in Kenya and Tanzania are operating as private businesses, potentially moving into the formalized private sector in the near future. Still, many of these initiatives retain social business characteristics and may well also formalize as CBOs.

Almost three quarters (72%) of all organizations are legally recognized. Nonetheless, informality is prevalent in the East African countries, where two-thirds of the initiatives are formalized in Kenya (65%) and less than half in Tanzania (46%). In Latin America most of the surveyed organizations are formalized (Argentina 94%, Brazil 100%, Nicaragua 80%). Nevertheless, in these countries there is also a large number of unorganized waste pickers, working autonomously in recycling. Furthermore, the organizations in Argentina and Brazil highlighted their struggle to maintain their legal documents up-to-date, since the process is time consuming and costly.

**Fig 2:**
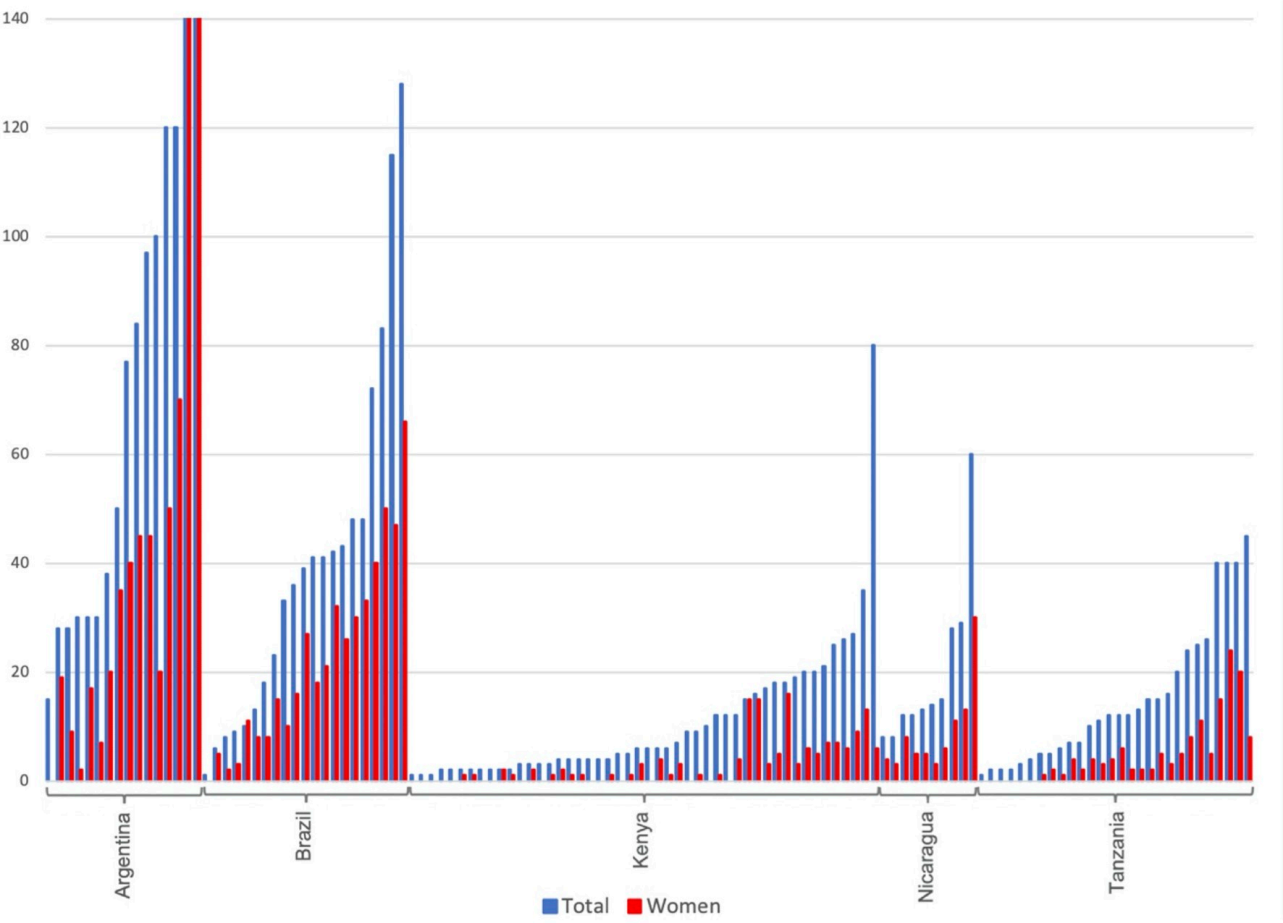
Number of participants in the initiatives, including gender composition. Number of members in blue; number of female members in red. Note that two of the initiatives in Argentina have significantly more members and end up outside the chart: one with 600 members of which 350 are female and one with 3,564 members of which 2,110 are female.

Around half of the Latin American initiatives have 40 or more members and some reach over 100 members (Fig 2). Smaller cooperatives have 10 to 20 members. The very large cooperative in Buenos Aires–Amanecer de los Cartoneros–was created by merging several WPOs in the metropolitan area. In Kenya, two-thirds of the initiatives have less than 10 members while just a few have 30 to 35 members. The same pattern is found in Tanzania, with the exception of two somewhat larger CSOs with 38 to 45 members. One Kenyan initiative, an informal organization of waste pickers at the waste dump in Kisumu, has 80 members and includes those (mostly homeless) who find their daily livelihoods foraging through the unsorted waste on the dump site.

**Fig 3:**
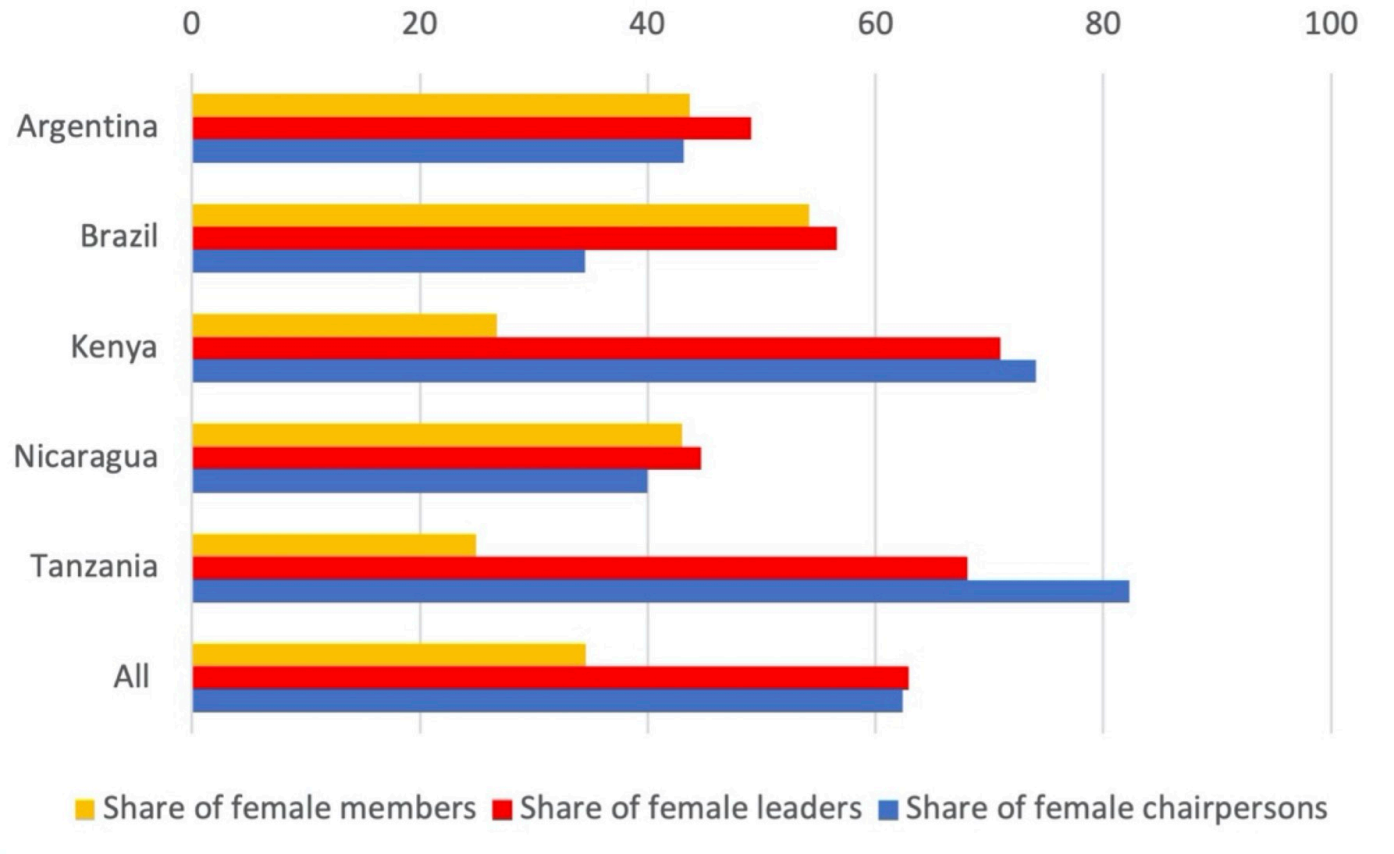
Gender of members, leadership groups, and chairperson (president or similar) in waste picker organizations. Expressed as share of females in %.

As indicated in Fig 2 there is a high level of participation of women in WPOs in both East Africa and Latin America. In the cooperatives in Brazil, the number of women is slightly larger than the number of men. In East Africa, less than a third of the members are women (Fig 3). When it comes to leadership, Latin American initiatives maintain an equal balance between women and men in leadership positions, while East African organizations are predominantly led by women. When it comes to the top leadership (chairpersons, presidents, etc.) the picture changes slightly. In Brazil there is a majority of male leaders but many initiatives also have a mixed top leadership. East African women are clearly at the head of an overwhelming majority of the organizations.

### Resources

Overall, Latin American initiatives have received more support from both governmental and non-governmental organizations compared to their East African counterparts (Table 1). Support was provided in initial stages of formation but there is also ongoing support in current activities. When it comes to the type of support provided as start-up, Argentinean and Nicaraguan initiatives obtained support in the form of funding and training whereas Brazilian WPOs received it as training and facilities (space, energy or water supply) but very little actual funding. In Brazil, other types of support include land from the municipality, equipment for sorting and transport, and computer equipment for management and infrastructure support is provided through grants from banks (e.g. Banco do Brasil) or state-owned enterprises (e.g. Petrobras). Other types of support in Nicaragua and Brazil mostly involved motorized and non-motorized vehicles for transport. In Tanzania there is less support and much of the funding constitutes minor sums of money provided by members themselves or by family and friends, sometimes as loans. Here, the capacity building is also fairly informal, provided by family or neighbours. Advice and financial support have further been provided by Chinese recycling businesses. In Kenya, a similar pattern with low levels of support and dependence on self-funding is present. Still, compared to Tanzania, there seems to exist slightly more support from governmental bodies, international funding agencies and NGOs.

**Table 1:** Numbers and types of support actions received by initiatives during start-up and currently.

Country	All support	Support to start the initiative					Present support (2018)				
	Quantity/ WPO	Funding	Training	Facilities	Other	Quantity/ initiative	Funding	Training	Facilities	Other	Quantity/ initiative
**Argentina**	3,3	31%	31%	25%	31%	1,2	25%	81%	56%	50%	2,1
**Brazil**	4,8	14%	90%	90%	76%	2,7	48%	62%	67%	29%	2,0
**Kenya**	1,3	27%	19%	13%	4%	0,6	19%	23%	15%	6%	0,6
**Nicaragua**	5,0	80%	90%	30%	80%	2,8	50%	50%	50%	70%	2,2
**Tanzania**	1,3	36%	39%	4%	14%	0,9	11%	7%	11%	4%	0,3
**All**	2,4	32%	43%	27%	28%	1,3	25%	36%	31%	20%	1,1

“Quantity/WPO” signifies the mean number of support types received by the initiatives but does not say anything regarding the volume of those support actions. Percentages signify the share of organizations receiving a type of support action.

Established initiatives in Argentina now receive more support compared to when they were start-ups, especially for training, but also facilities, working garments and equipment. Something to take into account is that much state assistance is delivered through wider social welfare programs rather than through specific measures targeting waste pickers’ demands. In Brazil, established WPOs continue to receive support, however, this now also includes funding from industry partners. Also, Kenyan initiatives manage to keep their support but at a continued low level, while established Nicaraguan and Tanzanian WPOs seem to have lost much of their initial support. In Nicaragua there were particular development-aid programs active for start-ups which are no longer running, yet current support includes equipment and advice from companies and NGOs. In Tanzania, a particularly important government support includes securing fee collection for waste collection and recycling to the benefit of WPOs.

WPOs in Latin America seem well connected with local, regional and national networks (Table 2). Most cooperatives in Brazil are linked to both regional networks and the national movement of waste pickers (Movimento Nacional de Catadores de Materiais Recicláveis, MNCR). In Argentina most cooperatives are linked to two national networks (Federación Argentina de Cartoneros, Carreros y Recicladores, FACCyR and Federación de Cooperativas Argentinas de Reciclado Autogestionadas, FECARA). East African initiatives are mostly connected only at the lowest local level and some do not mention any networks at all. Even if many Kisumu WPOs are part of a city-wide network (KIWAN), this network was inaugurated in 2018 and its resilience remains to be seen. Previous attempts of urban networking among Kisumu waste pickers have failed.

**Table 2:** Range of networking with other initiatives at local, urban, regional, national, and international levels.

	Range of networking			
Country	Local	Urban region	National	International
**Argentina**	63%	0%	88%	6%
**Brazil**	86%	86%	95%	5%
**Kenya**	44%	65%	0%	0%
**Nicaragua**	90%	0%	30%	0%
**Tanzania**	68%	14%	0%	0%
**All**	63%	43%	14%	2%

Networking is expressed as the percentage of initiatives engaging in the different types of networks.

### Activities

**Fig 4:**
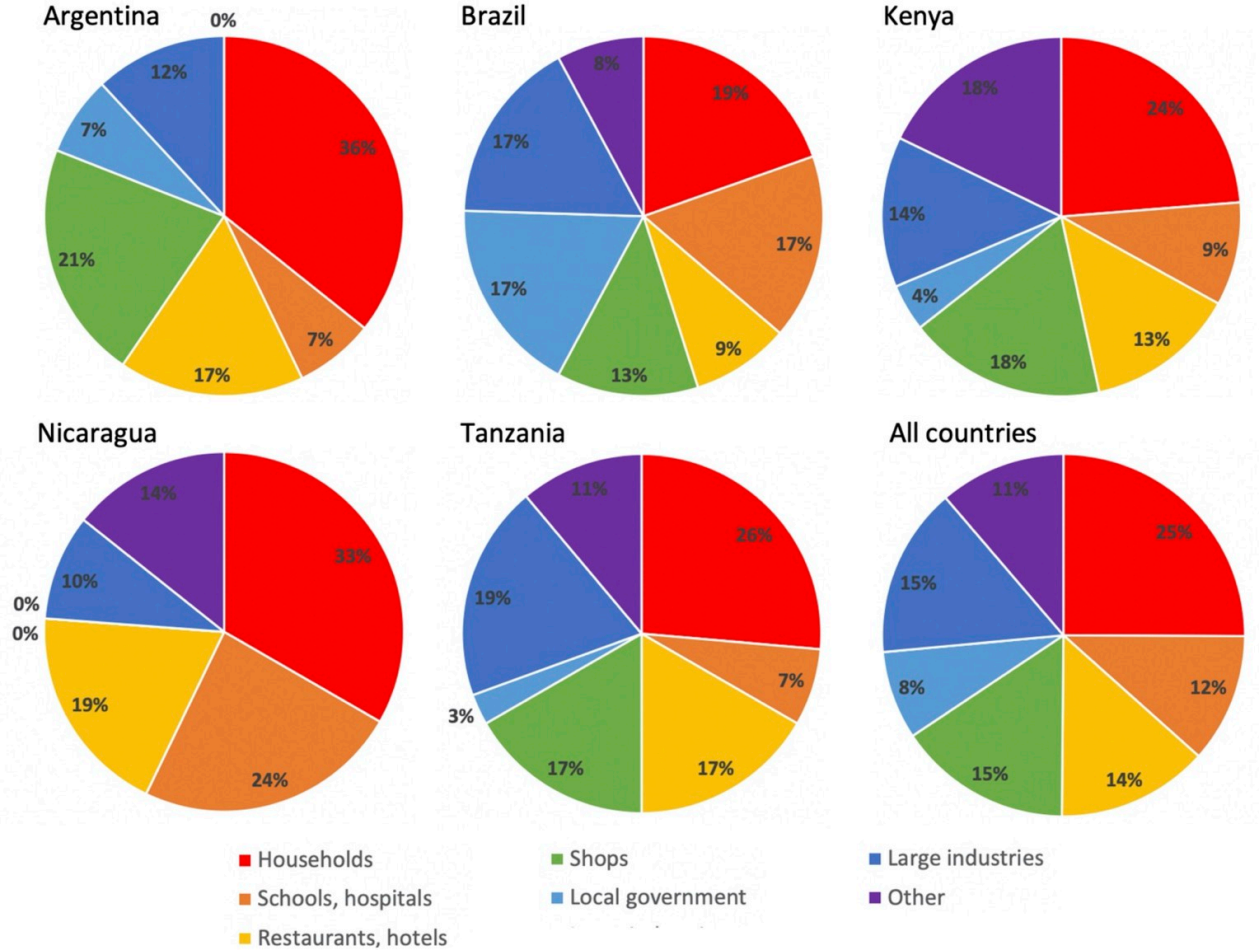
Types of clients. Divided into Argentina, Brazil, Kenya, Nicaragua, Tanzania, and All countries.

WPOs engage with many different types of clients (Fig 4). Argentinean groups have a comparatively large number of commercial clients, such as restaurants, hotels, shops and industries. Brazilian cooperatives also have many clients among public establishments, such as schools, hospitals and governmental institutions while in Nicaragua, local governments are not listed as clients. The East African initiatives share a similar distribution of clients and also here local governments are largely absent as clients.

In all countries households are the most prioritized clients but also industries are important (Table 3). In the similarly important “other” category, there is significant interaction and exchange between different waste pickers and waste entrepreneurs at the local level, especially in Kenya. When including second and third priorities, clients such as shops, restaurants, hotels, schools and hospitals emerge as essential customers. All in all, it is clear that the contribution of households towards the livelihoods and environmental services provided by waste pickers is significant [53] and that large industries also play a significant role, particularly in the case of Argentina and Brazil, while local governments have a remarkably low degree of engagement as clients in East Africa. The diversity of clients can be seen to measure the initiatives’ resilience and appears to be highest among Brazilian initiatives while groups in the other countries rely on fewer types of clients (Table 3).

**Table 3:** Prioritized clients.

	Prioritized clients				Diversity of clients Types of clients per initiative
	Prio 1	Prio 2	Prio 3	Prio 1+2+3	
**Argentina**	Households (81%)	Shops (31%)	Shops (19%)	Households (88%)	2.6
	Large industries (13%)	Restaurants, hotels (25%)	Schools, hospitals (13%)	Shops (56%)	
	Shops (6%)	Households (6%)	Restaurants, hotels (13%)	Restaurants, hotels (38%)	
		Large industries (6%)	Large industries (6%)	Large industries (31%)	
				Schools, hospitals (13%)	
**Brazil**	Households (57%)	Large industries (29%)	Schools, hospitals (14%)	Households (81%)	4.9
	Large industries (29%)	Households (24%)	Local government (14%)	Large industries (62%)	
	Schools, hospitals (5%)	Schools, hospitals (5%)	Large industries (10%)	Schools, hospitals (38%)	
	Shops (5%)	Shops (5%)	Other (10%)	Local government (24%)	
	Other (5%)	Other (5%)		Other (14%)	
				Shops (10%)	
				Restaurants, hotels (5%)	
**Kenya**	Households (48%)	Schools, hospitals (30%)	Schools, hospitals (20%)	Households (56%)	2.5
	Other (30%)	Households (20%)	Restaurants, hotels (20%)	Other (35%)	
	Restaurants, hotels (10%)	Restaurants, hotels (10%)		Restaurants, hotels (33%)	
	Large industries (10%)			Large industries (21%)	
				Shops (21%)	
				Schools, hospitals (15%)	
				Local government (2%)	
**Nicaragua**	Households (50%)	Schools, hospitals (30%)	Schools, hospitals (20%)	Households (70%)	2.1
	Other (30%)	Households (20%)	Restaurants, hotels (20%)	Schools, hospitals (50%)	
	Restaurants, hotels (10%)	Restaurants, hotels (10%)		Restaurants, hotels (40%)	
	Large industries (10%)			Other (30%)	
				Large industries (10%)	
**Tanzania**	Households (50%)	Restaurants, hotels (25%)	Shops (29%)	Households (68%)	2.6
	Large industries (32%)	Households (18%)	Restaurants, hotels (14%)	Large industries (50%)	
	Other (7%)	Other (18%)	Large industries (11%)	Restaurants, hotels (43%)	
	Schools, hospitals (4%)	Shops (11%)	Local government (7%)	Shops (43%)	
	Restaurants, hotels (4%)	Schools, hospitals (7%)	Schools, hospitals (4%)	Other (29%)	
	Shops (4%)	Large industries (7%)	Other (4%)	Schools, hospitals (14%)	
				Local government (7%)	
**All**	Households (54%)	Restaurants, hotels (19%)	Shops (9%)	Households (68%)	2.9
	Large industries (17%)	Households (13%)	Restaurants, hotels (7%)	Large industries (33%)	
	Other (16%)	Schools, hospitals (11%)	Schools, hospitals (7%)	Other (25%)	
	Shops (6%)	Large industries (11%)	Large industries (5%)	Shops (24%)	
	Restaurants, hotels (4%)	Shops (10%)	Local government (4%)	Restaurants, hotels (30%)	
	Schools, hospitals (2%)	Other (7%)	Other (2%)	Schools, hospitals (20%)	
		Local government (2%)	Households (1%)	Local government (7%)	

Expressed as a percentage of initiatives having clients as first, second and third priority. Clients with zero priority are not listed. The Prio 1+2+3 category is calculated by adding the percentages in the Prio 1, 2 and 3 columns. Note that not all initiatives have listed their second and third priority clients. The diversity of clients is expressed as the mean number of different types of clients per initiative.

When it comes to services offered, the collection of waste and/or recyclables is a priority in all countries, but in Kenya slightly less prominent (Table 4) due to the lack of tangible governmental support to WPOs. Looking across first, second and third priorities, recovery and sorting of recyclables is important in all countries, and in Argentina and Kenya also buying and selling of recyclables. In Argentina, buying from independent waste pickers is seen as an action of solidarity offering a better price to these vulnerable waste pickers compared to what is paid by middlemen, making them an attractive partner. This is a way to reach out to the informal waste pickers and encourage them to join a cooperative. In Brazil, in contrast, WPOs usually do not buy materials from independent waste pickers, middlemen, or larger waste generators. Since cooperatives in Brazil commercialize their materials on a regular basis and pay their members monthly, it would be difficult for them to pay independent waste pickers for their materials. The dependency relationship between autonomous waste pickers and exploiting middlemen remains very strong in Brazil.

**Table 4:** Prioritized services.

	Prioritized services				Diversity of services Types of services per initiative
	Prio 1	Prio 2	Prio 3		
**Argentina**	Collection (75%)	Sorting (50%)	Buying & selling (31%)	Collection (88%)	4.8
	Transportation (6%)	Buying & selling (19%)	Recycling (25%)	Sorting (69%)	
	Buying & selling (6%)	Collection (13%)	Sorting (13%)	Buying & selling (56%)	
	Sorting (6%)	Transportation (6%)	Processing (13%)	Recycling (38%)	
	Recycling (6%)	Processing (6%)	Education (13%)	Processing (19%)	
		Recycling (6%)		Transportation (13%)	
				Education (13%)	
**Brazil**	Collection (71%)	Sorting (76%)	Processing (48%)	Sorting (95%)	4.0
	Sorting (14%)	Collection (14%)	Buying and selling (38%)	Collection (86%)	
	Education (10%)	Processing (5%)	Sorting (5%)	Processing (57%)	
	Processing (5%)		Other (5%)	Buying & selling (38%)	
				Education (10%)	
				Other (5%)	
**Kenya**	Collection (42%)	Dumping/disposal (17%)	Dumping/disposal (17%)	Collection (48%)	3.0
	Buying & selling (35%)	Sorting (10%)	Sorting (10%)	Buying & selling (48%)	
	Sorting (6%)	Collection (6%)	Transportation (6%)	Sorting (40%)	
	Recycling (6%)	Transportation (6%)	Buying & selling (6%)	Transportation (31%)	
	Transportation (4%)	Buying & selling (6%)	Recycling (4%)	Recycling (21%)	
	Clean-ups (4%)	Recycling (4%)	Processing (2%)	Dumping/disposal (17%)	
	Other (2%)	Processing (2%)	Composting (2%)	Clean-ups (6%)	
		Composting (2%)	Education (2%)	Other (6%)	
		Education (2%)	Clean-ups (2%)	Processing (6%)	
		Clean-ups (2%)		Advocacy (6%)	
				Composting (2%)	
				Education (2%)	
**Nicaragua**	Collection (80%)	Sorting (40%)	Sorting (30%)	Collection (100%)	3.0
	Sorting (10%)	Transportation (30%)	Transportation (20%)	Sorting (80%)	
	Recycling (10%)	Collection (20%)	Recycling (10%)	Transportation (50%)	
				Recycling (20%)	
**Tanzania**	Collection (61%)	Sorting (46%)	Processing (21%)	Collection (64%)	2.5
	Sorting (11%)	Transportation (32%)	Dumping/disposal (18%)	Sorting (68%)	
	Other (11%)	Collection (4%)	Transportation (14%)	Transportation (54%)	
	Transportation (7%)	Processing (4%)	Sorting (11%)	Processing (25%)	
	Recycling (7%)	Recycling (4%)	Clean-ups (4%)	Dumping/disposal (18%)	
	Buying & selling (4%)	Education (4%)	Other (4%)	Other (14%)	
				Recycling (11%)	
				Buying & selling (4%)	
				Education (4%)	
				Clean-ups (4%)	
**All**	Collection (59%)	Sorting (42%)	Processing (15%)	Collection (67%)	3.3
	Buying & selling (15%)	Transportation (19%)	Buying & selling (13%)	Sorting (63%)	
	Sorting (9%)	Collection (9%)	Dumping/disposal (11%)	Buying & selling (33%)	
	Recycling (6%)	Recycling (6%)	Sorting (11%)	Transportation (30%)	
	Transportation (4%)	Buying & selling (5%)	Transportation (7%)	Processing (20%)	
	Other (3%)	Processing (4%)	Recycling (6%)	Recycling (17%)	
	Education (2%)	Advocacy (2%)	Education (2%)	Dumping/disposal (11%)	
	Clean-ups (2%)	Other (2%)	Clean-ups (2%)	Other (7%)	
	Processing (1%)	Education (1%)	Other (2%)	Education (5%)	
			Composting (1%)	Clean-ups (3%)	
				Advocacy (2%)	
				Composting (1%)	

a) First priority; b) Second priority; and c) Third priority. Services with zero priority are not listed. The Prio 1+2+3 category is calculated by adding the percentages in the Prio 1, 2 and 3 columns. The diversity of services is expressed as the mean number of different types of services per initiative.

Latin American WPOs typically engage in the collection of recyclables only, i.e. not of general household waste. In Brazil, the cooperative either collects the recyclables directly from the households or receive the materials from companies that provide selective collection services to municipalities. The cooperative then separates and sells these recyclables either individually or collectively with other cooperatives. Some Argentinean and Brazilian cooperatives also engage with industries under frameworks for Expanded Producer Responsibility (EPR), such as reverse logistic agreements and corporate environmental auditing (e.g. with ABIHPEC, the Brazilian association for packaging of hygiene products) [see also 13, 54]. WPOs provide a service to these industries by managing their recyclable waste, helping them to keep up with their EPR requirements and allowing them to reach recycling targets and to accredit their green metrics through transfer of proof of recycling compliance.

Collecting and transporting waste is a rather prominent activity in Kenya, Nicaragua and Tanzania. Collecting and dumping and disposal of waste is a common waste picker activity in both East African countries, but are absent among Latin American initiatives. This fact reflects the absence of public waste management in East Africa. Although processing and recycling (upcycling) are not listed among the main priorities, they still constitute key services in all five countries apart from Nicaragua, where the lack of infrastructure and technical capacity restrict this activity. Education, public clean-ups and advocacy appear to be of little importance in most cases. In Brazil and Argentina, WPOs sometimes engage in educational activities involving schools or community centres. Collecting organic waste for composting is an even less prominent service, which could be significant given the predominance of the organic fraction in household waste in this part of the world [25, 55]. Regarding the diversity of services (Table 4), Brazil and especially Argentina stand out, while initiatives in the other three countries appear more specialized. Many initiatives in Kenya and Tanzania engage in just one or two types of services, making them vulnerable to fluctuations in demand.

When it comes to recycling, Argentinean and Nicaraguan initiatives focus on paper, cardboard, plastic, metal and glass (Table 5). Brazil is similar but also includes white paper and multilayer Tetra Pack as main items. In Argentina and Brazil, and to some extent also in East Africa, each category includes a great variety of subcategories; e.g., plastic is separated into high density polyethylene (HDPE) and polypropylene (PP), low density polyethylene (LDPE), polystyrene (PS), and polyethylene terephthalate (PET, sorted into three different colours), as well as soft plastics sorted into transparent/black, clean and dirty; each one of these fractions having specific recycling destinations and prices. In Argentina and Brazil, most of the recovered plastics feed local industries and manufacturers, rather than being exported abroad as is usually the case in East Africa. Both Kenyan and Tanzanian groups primarily focus on plastic and metal, with paper as a third main material, and much goes for export. Comparing the two continents, the market for different types of recycled paper seems stronger in Latin America while in East Africa plastic and metal are the main resources generating income.

**Table 5:** Types and share of recycled materials.

	Argentina	Brazil	Kenya	Nicaragua	Tanzania	All
**Share of recyclables**	Paper (20.3%)	Plastic (16.9%)	Plastic (30.1%)	Paper (20.5%)	Plastic (34.8%)	Plastic (24.7%)
	Cardboard (20.3%)	Paper (14.6%)	Metal (21.5%)	Cardboard (7.7%)	Metal (19.6%)	Metal (18.2%)
	Plastic (20.3%)	Cardboard (14.6%)	Paper (14.0%)	Plastic (25.6%)	No recycling (13.0%)	Paper (15.8%)
	Metal (17.4%)	White paper	Organic waste	Metal (20.5%)	Paper (10.9%)	Glass (11.9%)
	Glass (11.6%)	(13.5%)	(8.6%)	Glass (25.6%)	Glass (8.7%)	Cardboard (10.4%)
	General recyclables(4.3%)	Metal (13.5%)	Glass (6.5%)		Cardboard (6.5%)	White paper (3.9%)
	Textile (4.3%)	Glass (13.5%)	Bones, fish scales (4.3%)		General recyclables(2.2%)	General recyclables(2.7%)
	Tyres, rubber (1.4%)	General recyclables* (4.5%)	Cardboard (2.2%)		Organic waste (2.2%)	Organic waste (2.7%)
		Newspaper (1.1%)	Textile (2.2%)		Electronics (2.2%)	Electronics (2.7%)
		* Includes cooking oil, fluorescent lamps, etc.	Charcoal dust (2.2%)			No recycling (2.1%)
			General recyclables(1.1%)			Textile (1.5%)
			White paper (1.1%)			Bones, fish scales (1.2%)
			Electronics (1.1%)			Tyres, rubber (0.6%)
			Tyres, rubber (1.1%)			Charcoal dust (0.6%)
			Saw dust (1.1%)			Saw dust (0.3%)
			Furniture (1.1%)			Furniture (0.3%)
			Ash (1.1%)			Newspaper (0.3%)
			No recycling (1.1%)			Ash (0.3%)
**Diversity of recyclables**	4.3	4.2	1.9	3.9	1.4	2.7

The diversity of recyclables is expressed as the mean number of different types of recyclables per initiative.

Another observation is that while Nicaraguan groups focus on just five materials (Table 5), Kenyan collection is, in comparison, much more diversified by including a large variety of items into their recycling, including a comparably large share of organic waste, saw dust, fish process residuals, to mention a few. In Tanzania and Kenya, groups collect household waste without engaging in recycling, something that is not visible in the other countries. Furthermore, although some East African groups work with two to five categories of recyclables, many focus on just one category. While a larger diversity of materials would lead to greater resilience in the informal waste sector, widespread poverty and long distances to buyers often force groups to identify small niches of recyclables that can generate at least a minimum revenue. It is important to note that some WPOs have difficulties with maintaining the uniformity of collected materials, which then restricts their access to a wider market. In Argentina and Brazil, glass, tetra pack and fabrics are often not collected by waste pickers due to oligopolistic and regionalized market conditions imposing such low prices that it is not worthwhile to collect and sort those materials, even though they are technically possible to be recycled. Such market deficiencies leave the WPOs with collected recyclables, which then involve costly dumping fees. In Brazil, for example, cooperatives collect and separate multi-layered packaging (such as aluminium/plastic snack packages) which do not have a market for recycling and which takes up their time, storage and ultimately costs money to dispose.

### Challenges and innovations

WPOs face many challenges in both their formation and operation (Table 6). Lack of capital is a major hurdle to improve and expand operations or to become formalized. Price fluctuations for recyclable materials, dependency on intermediaries or specific industries as buyers for their materials, high competition with large waste management and recycling companies and other WPOs, were also stated as major threats in all countries. Some groups mentioned that operating in deprived neighbourhoods with low-income residents and low rates of payment added further challenges, as in these localities the ability to pay for services is low, even if the will is there. Barriers in the commercialization of materials due to lack of initial knowledge of retailers and the supply chain, and thus having lower bargaining power, were mentioned by many groups. Groups that were better connected suffered less from such knowledge shortfalls, e.g., those groups in Brazil that were connected to a regional network. Lack of recognition and policies, prejudice and stigma, and persecution of waste pickers and groups still are major issues for many groups across the studied geographic spectrum. Enclosure of landfills (as in Managua) and restrictions and partial closure of dump sites (as in Kisumu) have made survival particularly difficult for waste pickers.

**Table 6:** Challenges perceived by waste picker organizations.

**Resources**	Lack of initial capital and capital to grow, lack of trust of financial institutions to get loans.
	Machines donated often are not the solution: do not fit local requirements and are abandoned or break down.
	Lack of necessary facilities (storage, causing environmental pollution), transportation, machines, tools.
	Formalisation, lack of official documents (certifications, permits).
**Market**	Commercialization (initial lack of knowledge of retailers, sales of materials, supply chain, low bargaining power, market price fluctuation, low profit rates).
	Lack of technical, political, legal or financial incentives to expand the range of recycled materials.
	Competition both between groups and with large companies, including the threat of large-scale incineration.
	Waste collection customers in low-income settlements are not paying.
**Operation**	Precarious working conditions, high member turnover, governments not honouring service contracts.
**Legislation**	Legislation/illegality (impeding certain activities), polyethylene bag ban in Kenya (withdrawing a recyclable), police persecution, harassment, bribes.
**Management**	Internal conflicts, lack of trust, lack of group cohesion, lack of experience in administration, conflicts in leadership, bad leadership, bad management, absenteeism, lack of transparency, culture of working solo and lack of experience of collective management. The same individuals tend to remain in leadership positions.
**Social**	Insufficient inclusion of women.
	Unequal distribution of benefits, funds.
	Conflicts within and between groups.
	Alcoholism.
**Environmental**	Lack of environmental awareness (illegal dumping).
**Knowledge, identity**	Knowledge and capacities (e.g. to treat machines, to reach retailers).
	Lack of advocacy skills.
	Stigmatisation and prejudice, specifically associated with child labour.
	Society’s lack of knowledge about waste pickers and waste.
	Animal cruelty.

Many groups mentioned internal management challenges connected to lack of trust among members, lack of transparency, insufficient knowledge of management, and challenges that come with transitioning from an individual and autonomous way of conducting business to being part of a collective culture. Such challenges are also present in the metropolitan, regional and national networks. The absence of technical, political, legal or financial incentives to expand the range of recyclable materials worth recovering and commercializing were other key aspects that resurfaced in many interviews. Particularly in Brazil, Argentina and Kenya the threat of the introduction of incineration which would harm recycling practices was mentioned by some groups.

Despite the many challenges, the achievements of waste picker initiatives are many. A good number of the WPOs are innovative in terms of technology and product development, commercialization, management, alliances and governance, social improvements, co-creation of knowledge, and formation of identity (Table 7).

**Table 7:** A summary of identified innovations through interviews by country and type of innovation.

	Argentina	Brazil	Kenya	Nicaragua	Tanzania
**Technology and products**	Research and Development on non-marketable recyclables (design of processes and machinery to transform non-marketable plastics and cellulose materials into marketable recyclables). Reciplazas, children playgrounds furniture (more quality, durability and aesthetics than standardized production).	Identification and processing of new materials. Factory of polymer and collection of cooking oil to be converted into products (fuel, soap, etc.).	Processing materials (e.g. reuse of charcoal dust in briquettes). Processing machines for added value and transformation of materials (bailing machine). New products (e.g. plastic fencing poles out of polyethylene bags, woven bags, mats and cushions). Transportation means (more hand carts).	New products (jewellery).	Processing machines for added value and transformation of materials (e.g. crushing machines). Transportation means (e.g. compressor trucks). Identifying collecting new materials (e.g. e-waste).
**Commercialization**	R&D about non-marketable materials allows to add value to those materials (Expanded polystyrene, Doy pack and beer labels) which otherwise would not have value, destined for the landfill. They are key to validate the waste picker cooperatives as professional service providers to large manufacturers, as they address the "cradle-to-cradle" traceability not only of regular ‘recyclables’ (cardboard, PET, paper) but also for ‘non-marketable’ waste, which otherwise is landfilled.	Collective commercialization between networks of waste picker cooperatives. Floating capital to enable collective sales. ‘Reciclagem Popular’, a method employed by the National Waste Pickers Movement (MNCR) where waste pickers are the teachers and through popular education teach about how to control the recycling chain, collection technology and the organization of waste pickers. Partnerships with companies and industry associations (e.g. ABIHPEC, PEPSICO, SESC).	Community clean-ups (as a marketing and educational tool). Using youths for door-to-door sensitization. Diversification of services (e.g. cleaning toilets in Nairobi in partnership with CCS, car washing, pit and septic tank emptying). Engaging landlords in waste collection. Training hotels (street food restaurants) to sort out waste. Marketing and social media. Linkages with waste collection networks to obtain recyclable materials to better price.	Partnerships between large beverage corporations for waste collection of e.g. glass, as part of a reverse logistics system.	Selling to larger retailers. Partnership with companies (e.g.Soyana). Locating operations in untouched markets (far from the city). Provide a regular collection service. Payment system through bank account, EFD machine (avoiding un-payment). Educational material for customers (flyers).
**Management**	Official contracts between coops and companies, backed by the environmental authorities.	Participatory decision making, self-management, transparency and full access to all information by all members. Autonomy.	Training in bookkeeping, team building, group management.	Internal management, learning collective interests, unity, self-organization.	Distributed leadership, participatory management (e.g. UWAWABU community group), shared management with the local government whenever waste pickers were provided contracts by the government.
**Alliances and governance**	Alliance with NGOs and authorities.	Contracts between local government and waste picker cooperatives (e.g. Ourinhos, Mauá, Ribeirão Pires) for selective waste collection. Recognition and remuneration of the service provision. Conversations and technical support with other recycling groups and networks.	Training and capacity building in partnership with NGOs, universities and governmental agencies. Partnering with county government and city authority for transportation to the dumpsite	Partnership with local government and private companies (e.g. waste transportation by boat).	Alliances with formal small and medium-sized enterprises in providing transportation and other services.
**Social**	Redefinition of which materials are socially considered ‘recyclable’ and which not. Creating products to improve low-income neighbourhoods, children and people with disabilities.	Creating low barrier work opportunities. Workers’ health improvement and risk reduction.	Improving financing opportunities through table banking. Generating incomes for unemployed youth. Providing protective equipment (boots, gloves) for waste pickers.	Generating income for women.	Offering lunch, food, accommodation, loans for members. Providing jobs for women, especially widows.
**Knowledge and identity**	The Research and Development praxis on this material involves a complex knowledge co-production, which is derived from waste pickers themselves.	Support and capacity building (e.g. accounting) through Instituto Catasampa and Rede Cata Vida. Training programs supported by MNCR, NGOs (e.g. Gaspar Garcia, ProjetoBrasil-Canada), government funded programs (Cataforte, SENAC) etc. Training and competences lead to empowering.	Self-learning (identifying products and markets). Learning a profession: materials, supply chain, markets. Partnership with NGOs for training and capacity building.	Mapping and surveying waste pickers and WPOs, as part of a recruitment process by the Nicaraguan Waste Picker Organization RedNica. Punctual training as a result of aid-development programs supporting WPO start-ups.	Training members in customer service. Overall training on business management and operations of waste management.

Examples of *technological grassroots innovations* have emerged from many groups who have set up processing machines for transforming materials to add value or for producing new materials, e.g. turning charcoal dust into briquettes and plastics into fence posts in Kenya; children playgrounds equipment (*Reciplazas*) in Argentina; and jewellery production from recyclables in Nicaragua. On a more advanced level in Brazil, the cooperative Coreso, together with 13 other groups, has formed the network Rede Solidária Cata-Vida, running a polymer processing unit and a processing plant for cooking oil.

Innovative *commercialization* is closely linked to technological and product innovation. In Argentina, the cooperative Reciclando Sueños, together with university researchers, has created Research and Development (R&D) projects to transform non-marketable recyclables, currently rejected by the local recycling industry and still dumped in landfills, into marketable materials. Successful examples from this cooperative of materials now possible to recycle include expanded polystyrene, multi-layered plastics and beer labels. Such development of knowledge and competences becomes key to strengthen waste pickers’ role as waste management service providers to large manufacturers. Among Brazilian WPOs, novel modes of collective commercialization and partnerships have made operations more resilient, increasing income and visibility of the cooperatives. In both Kenya and Tanzania, much effort is placed in multiple ways of sensitization to build markets, where community clean-ups as marketing and educational tools are a typical approach. Argentinean, Brazilian and Nicaraguan WPOs engage in reverse logistics agreements with large corporations for their compliance of environmental legislation. Also Tanzanian WPOs partner with large companies and retailers, but here the reverse logistic component is still missing.

When it comes to *management*, innovation is often closely linked to the origins of many WPOs in different types of social and political struggles. Distributed leadership, participatory decision making, self-management, and transparency for all members regarding both information and bookkeeping are key elements in all five countries (but not without problems, as mentioned in the challenges section). It was indicated that strengthening women participation is particularly relevant in Brazil and Nicaragua. Management is also linked to the different types of novel *alliances*, partnerships and contracts with local authorities, private companies, NGOs and universities. Through these, WPOs seek to position themselves as key actors in the *governance* of the local waste management system.

In Tanzania, a number of *social* innovations extending benefits such as meals, accommodation or loans for employees are included as part of corporate social responsibility (CSR) in the partnerships between informal waste pickers and small companies involved in the waste management supply chain. In Kenya, due to the prevalent lack of support from authorities, the social and solidarity dimension of waste innovations is significant, including self-financing schemes, job-generation and improvement of health among the most excluded waste workers.

Significant advancement of *knowledge* and skills in collecting, sorting and recycling is another innovation. In Argentina, Brazil and Nicaragua, development of competences becomes key to strengthen WPOs’ role as waste management service providers to large manufacturers. By so doing, as described above, WPOs can guarantee traceability for the reverse logistics of recyclables (e.g. cardboard, PET, paper) as well as initiate recycling of hitherto non-marketable fractions. In Kenya and Tanzania, training is generally provided in collaboration with NGOs but also as part of (often informal) mentorship programs carried out within communities. Although preconditions and ambitions differ, the various initiatives for knowledge development among waste pickers clearly have a strong influence on their *identity* as crucial providers of environmental services, as seen both by themselves and by society at large.

## Discussion

To examine the characteristics, challenges and innovations of WPOs, the discussion is structured into two areas: organisational and operational characteristics; and challenges and innovations.

### Organisational and operational characteristics

First, institutional arrangements, including innovations in legislation, public policies and governance approaches, to a large extent determine how WPOs develop. Already existing organisational structures explain the predominance of more collective forms of organization (such as cooperatives) in Latin America [56]. Investments by progressive official leaders, negotiated and achieved by the cooperative sector via its solidarity economy and supported by poverty eradication programs in Brazil, have allowed WPOs to access needed resources and legislation in support of their activities [57]. A more neoliberal context in Kenya supports entrepreneurship as an operational format [36]. At the same time, both East African countries have a tradition of self-organized women groups, youth groups and table banks rooted in African institutions of community organisations [58].

Second, while in many countries the number of WPOs has increased over the recent years, we observe a high turn-over in membership. This is particularly the case where the WPOs are linked to precarity during economic recessions, with more people engaging in waste collection as an ultimate livelihood option [59]. East African WPOs are often smaller in size, being community-based or stemming from such organizations. The bigger size of WPOs in Latin America reflects how more sophisticated organisations have succeeded to grow, stabilize and create nested organisational structures with different levels of organisation, such as associations of cooperatives and regional/national networks in Brazil [60], or the creation of federations in Argentina [61]. In both Argentina and Brazil, regional/national WPOs have played a key role in changing public waste management policies, turning exclusion and lack of recognition into a “troubled collaboration” through a combination of contention and collaborative relations [62]. Some authors still warn about the risk of certain grassroots innovations just providing band-aid solutions, thus even reinforcing existing social and economic inequalities in the long run [29].

Third, previous studies have found that women tend to be excluded from leading positions in more formalized WPOs while they have more clout in community-based WPOs [63]. While the data from this study seem to confirm this observation in relation to less formalized and small-scale WPOs in East Africa, the Latin American organizations are still far from a 50/50 division of leadership between genders. Our findings support the statement that WPOs have a significant potential for supporting the SDG for gender equality, as well as many other objectives covered under this agenda [33, 64]. Nevertheless, Vasconcellos, Dias and Fraga [38] warn about the visible and invisible gender biases that may undermine the role of women within innovation processes. In Latin American WPOs, these risks seem to be present, with clearly less than 50% of the leadership being women, and particularly in the Brazilian national waste pickers movement (MNCR) most leadership positions have been occupied by men for more than a decade.

Fourth, the more organisational levels, tiers and connectivity a WPO has, the more it can exert influence on the context. In Latin America, many neighbourhood WPOs (cooperatives, associations, CBOs) have expanded into community and city-wide or even regional networks to stimulate collective actions, including commercialization and negotiations among cooperatives and with governments [16]. In Argentina and Brazil, such networks have flourished to support the exchange of experiences, policy influence and other necessary resources among WPOs, and perform collective commercialization of recyclables, cutting out the middlemen to increase revenues and livelihood security. Complex networks of WPOs, often supported by civil society organizations, practitioners and activists have been able to generate bottom-up and innovative waste solutions that involve the resources, knowledge, interests and values of local communities [29, 34].

Over the past decades, national waste picker movements have been established in several Latin American countries, such as FACCyR and FECARA in Argentina, MNCR in Brazil and RedNica in Nicaragua. WPOs in any context can benefit from peer-to-peer learning regarding benefits and detriments of different organizational set-ups. Our Brazilian and Argentinean cases demonstrate the power of peer-to-peer knowledge sharing, characterizing cooperatives, networks and federations as important innovation spaces for sustainability and social justice [65], fighting the still prevailing epistemic asymmetries [42]. Specifically, MNCR in Brazil and the regional social networks in Argentina have played crucial roles in building the capacities of waste pickers and providing them with access to knowledge and information that helps expand the activities of the sector.

While the Brazilian MNCR is well established, and the FACCyR has increased its role in shaping public waste management policies, the RedNica represents an intermediate situation, struggling to support the creation and stabilization of WPOs with almost no support from the government. Waste pickers have also been organized across the continent through the Latin American Waste Pickers Network (RedLACRE) and globally in the Global Alliance of Waste Pickers. These national/global waste pickers federations and movements have been important to prompt societal awareness of rights and demands of informal workers, and to strengthen their political influence through collective action [4]. Especially the cases of Brazil and Argentina highlight how institutional and legal structures are crucial to support the creation and further development of WPOs. The Brazilian solid waste legislation [40], elaborated with the input from MNCR, has promoted WPOs and provided financial support to build their skills, equipment and infrastructure through a solidarity economy framework. Through this, the activity of waste pickers is now recognized as a profession [16, 66].

The situation in East Africa is different. WPOs are well connected at the very local level with residents of informal settlements not serviced by municipal waste collection services, but have difficulties in accessing authorities and markets for recyclables. Here, the more recent city-wide WPO network Kiwan in Kisumu carries a potential to bring benefits also for Kenyan waste pickers [4]. Governmental arrangements for the co-production of waste collection services between WPOs and the city (e.g. agreements for remuneration of waste pickers, for regular evacuations of transfer points or licenses to operate) depend on well-established and long-term relationships, with networks and partnership arrangements integrated in transparent and inclusive governance structures [10, 67], and with policies and national legal frameworks in place [68].

### Challenges and innovations

First, our results corroborate previous research reviewing how a combination of policy and legal concerns, organisational challenges, as well as financial, social and technical issues shape–and often hinder–WPOs’ ability to provide waste services and improve living conditions among waste pickers [29, 34, 68, 69]. The findings show how it takes both time and continuous and reliable support to stabilize WPOs by making it possible for them to grow in numbers, confidence, revenues, knowledge and networks. WPOs occupy “niches” outside of the mainstream systems, shaped by social inequalities and deeply rooted asymmetries and vulnerabilities, which affect the accomplishments and failures of these innovations and how they become acknowledged (or not) [40]. The cases in Brazil and Argentina illustrate how institutional support is fundamental and how the lack thereof often threatens their existence. Although WPOs need to be both resourceful and self-reliant, the positive impacts of long-term support go beyond start-up assistance and instead create a leverage on institutional conditions for WPOs to grow. Such support considerably improves the delivery of waste services to the many unserved residents in informal settlements, at the same time offering dignified working conditions for the waste pickers. This finding is in line with Tirado-Soto and Zamberlan’s research on the creation of networks of waste pickers’ cooperatives in Brazil, where WPOs typically need “more time to become cohesive and organic” [12].

Second, in Brazil, the Social Economy Movement and the Social Technology Network Movement have been instrumental, not only in the creation and support of cooperatives [70, 71], but also in the development of legal frameworks, such as the law of “reverse logistics” [16, 72]. Even if waste cooperatives are paid by corporations for the quantity of materials recovered, this payment is often in the form of infrastructure investments and not cash, a system that maintains paternalistic relationships between government or industry and the WPOs [73]. Also in Argentina, new provincial regulations in Buenos Aires have opened up for WPOs to develop grassroots innovations and through these provide new recycling services to large generators (manufacturers, malls, private urbanizations), here being paid in cash for the amount of tonnes they divert to the recycling industry [42, 45]. As Dias has shown, organizing through WPOs “provides an avenue for political action that can lead to transformative changes at a national level”, such as the abovementioned legal frameworks that “recognize waste pickers’ legitimate access to waste” [15].

Third, the creation of WPOs in itself represents a major environmental grassroots innovation, created out of “nothing” [74] due to WPOs’ abilities to find and mobilize resources in contexts of scarcity and uncertainty by bootstrapping [75] and bricolaging [6, 35, 36]. Many WPOs, particularly those in Tanzania and Kenya, are deeply woven into the territorial and commercial relations of trust and reciprocity in the informal settlements they serve and where they reside [36, 76], drawing on crowdfunding or table-banking infrastructures to pool savings and mobilize scarce resources among poor relatives, friends and neighbours for seed capital to start up waste picking activities [58]. In Kenya and Tanzania, the entrepreneurial developmentalism paradigm [77] promoted by aid development organisations has resulted in the substitution of many self-help groups with microentrepreneurs and in the consolidation of public-private partnerships. The risk of such policies, strongly aligned with neoliberal agendas, is that they may miss out on the lessons learnt from Latin America linked to sectoral collective action strategies [61], to the detriment of the development and resilience of East African WPOs. Another example of development aid harmful to WPOs is found in Managua, where the municipality displaced hundreds of waste pickers from the municipal landfill and created a municipal recycling corporation that employed around 700 former waste pickers, but that disconnected even more of them from their source of livelihood [24].

## Conclusions

Grassroots WPOs contribute to the economic, social and environmental sustainability of the societies they serve. They are incredibly important for cities to move to sustainability and beyond by *performing* sustainability, not only in informal settlements, but also by providing services tackling the consumption and production processes of their wider urban populations. Selective waste collection, separation and diversion into recycling are crucial to maintaining cities clean and to fuel the recycling industry, where interruptions affect both public health and the local economy. The contributions of WPOs can also be expressed through the Sustainable Development Goals, where WPOs tackle several of these goals [64]. By acknowledging these contributions and by involving WPOs in the evolution of formal waste management services and urban service provision in general, the positive impacts of WPOs and similar organizations in other types of urban service provision would mushroom.

To start and maintain WPOs in informal settlements is a difficult quest, given the lack of infrastructure, institutional frameworks, and public or private investors. Despite operating in resource-poor and turbulent environments, WPOs show how their resilience draws on and shapes combinations of characteristics and dynamics, such as size; history of the initiative; connectivity through networks and governance tiers; diversification of operations, activities, materials and customers; and the historical pathways of local and national policy and legal frameworks. Experiences from Latin American cooperatives illustrate the successful management of these factors to become increasingly grounded in institutional settings and public support. The activities of different types of networks and support organizations in Brazil and Argentina show the important role also of intermediary actors to mobilize necessary resources, including knowledge and legal frameworks. In East Africa, the findings have highlighted different entrepreneurial models and their abilities to deliver waste services and develop livelihoods in contexts of widespread scarcity. These entrepreneurial models do not rely on the type of networks seen in Latin America, but on markets where small-scale investors provide capital to turn small CBOs into companies that can connect to and incorporate market structures for their logistics and commercialisation. Still, such transitions also expose them to the dire competition from other companies. Instead of being sustained by supporting local social networks, they gradually drift away from the community and strong connections to local contexts.

This article provides a description of the rich organisational and operational characteristics of WPOs and identifies a wide range of taxing challenges and effective innovations developed by WPOs. The different models in Latin America and East Africa have their strengths and weaknesses in relation to the complexity of local realities. Complementary and comparative research in other WPO contexts is needed, for example regarding pros and cons of various models of grassroots service provision; learning how various actors and institutions interacting with WPOs and their grassroots innovations should engage and connect; and how the self-sustainability and resilience of WPOs can be strengthened and maintained. Furthermore, we wish to propose a context sensitive South-South and practice-policy-research learning across different models for grassroots service provision, to take full advantage of what has been experienced, innovated and delivered in the many informal settlements of the Global South. Bringing such cross-learning to fruition then depends on the initial and sustained support provided to WPOs and other types of informal service deliverers by diverse governmental, institutional, sociocultural and market structures. Sustaining WPOs as important providers of socio-environmental benefits through governmental and non-governmental actions pays back manifold.

## Supporting information

S1 Appendix Recycling networks mapping waste governance survey. (DOCX)

S2 Appendix Recycling networks mapping waste governance interview guide. (DOCX)
