# Supplementary material for: Characteristics, challenges and innovations of waste picker organizations: A comparative perspective between Latin American and East African countries
Source: PLoS One. 2022 Jul 29;17(7):e0265889. doi: 10.1371/journal.pone.0265889 (PMC9337677; doi:10.1371/journal.pone.0265889)
Supplement: S1 Appendix — (DOCX) [file pone.0265889.s001.docx]

***Recycling networks***

***Mapping Waste Governance***

***Survey***

Name of Respondent: ______________________ Survey No: ___________________________

Name of the initiative: ______________________ Location: ____________________________

Your position within the initiative: ______________________________________________________

Your contact information: _____________________________________________________

Name of researcher: __________________________________________Date: ____/______/______

**I. Background**

1. Starting date of your initiative:
2. Is your initiative a:

- NGO
- Civil society organization (CSO)
- Cooperative
- Association
- Self-help group
- Private company
- Religious based organization
- Not yet registered
- Other, explain: …………………………………………………..

1. Are you legally recognized?

- Yes. Since when?........................................
- No

1. What are the requirements for recruiting members/employees? _______________________
2. Total number of participants in the initiative: ______
3. What is the gender composition of?

|  | Women | Men |
| --- | --- | --- |
| Initiative as a whole |  |  |
| Directive ,commissions, leaders |  |  |
| President /Chairperson/ |  |  |

1. Did you received any kind of support to start the initiative? Please specify of what kind, who is supporting and quantity

| Funding |  |
| --- | --- |
| Training |  |
| Facilities |  |
| Others |  |

1. Do you receive any kind of support nowadays? Please specify of what kind, who is supporting and quantity

| Funding |  |
| --- | --- |
| Training |  |
| Facilities |  |
| Others |  |

**II. Collection, processing and commercialization**

1. Describe the services you provide and prioritize the three most important

| Mark with X | Prioritize (1, 2, 3) | Services provided | Specify |
| --- | --- | --- | --- |
|  |  | Waste collection |  |
|  |  | Waste transportation |  |
|  |  | Dumping or disposal |  |
|  |  | Buy and selling |  |
|  |  | Sorting |  |
|  |  | Processing |  |
|  |  | Recycling |  |
|  |  | Composting |  |
|  |  | Educational services |  |
|  |  | Clean-ups |  |
|  |  | Advocacy |  |
|  |  | Other |  |

1. Identify your clients, prioritize them, and describe type of agreement and main services provided to those clients

| Mark with X | Prioritize (1, 2, 3) | What kind of agreement?  (contract…) | Client | Main services |
| --- | --- | --- | --- | --- |
|  |  |  | Households |  |
|  |  |  | Schools, hospitals |  |
|  |  |  | Restaurants, Hotels |  |
|  |  |  | Shops |  |
|  |  |  | Local government |  |
|  |  |  | Large industries |  |
|  |  |  | Other |  |

1. What type of materials do you collect?

- Mixed household waste
- Mixed market waste
- Recyclable market waste (dry). Specify_______________________
- Recyclable household waste (dry) Specify ____________________
- Organic market waste (humid)
- Organic household waste (humid)
- Industrial waste. Specify:
- Commercial waste (offices and super markets). Specify
- Institutional waste (hospitals, schools. Specify:

1. What transportation mode do you use for collection and commercialization? Ownership?

|  | Collection | | Commercialization | |
| --- | --- | --- | --- | --- |
|  | Owned | Rented | Owned | Rented |
| Hand pushed cart |  |  |  |  |
| Horse pushed cart |  |  |  |  |
| Bicycles |  |  |  |  |
| Motorbike with cart |  |  |  |  |
| Motor vehicle |  |  |  |  |

1. What is the location for your operations? (You may choose several and explain)
   - - Landfill
     - CBD or commercial areas
     - Market places
     - Formal neighborhood
     - Informal neighborhood
     - Recycling center/cooperative
     - If other, explain: ……………………………………………………………………………
2. Describe infrastructure at your working space and ownership:

|  | Rented | Owned | Access allowed |
| --- | --- | --- | --- |
| Uncovered space/open air |  |  |  |
| Partially covered installation |  |  |  |
| Covered permanent installation |  |  |  |
| Storage facility |  |  |  |

1. Accessibility to water, electricity and sanitation services at your work place

|  | Very accessible | Accessible | With difficulties | Not accessible |
| --- | --- | --- | --- | --- |
| Clean water |  |  |  |  |
| Electricity |  |  |  |  |
| Toilet/sanitation |  |  |  |  |

1. What equipment and what tools do you use?
   - - Conveyer belt
     - Table
     - Press
     - Balance
     - Mill
     - Other, explain: ……………………………………………………
2. Do you have storage facility available?

- Yes
- No

1. Which safety facilities do you have?

- Uniform
- Gloves
- Protection mask
- Special shoes
- Others, specify; ………………………………………………………………………………….

1. How do you sell the material? Specify %, and whom
   - Individually:
   - Collectively:
   - Other, explain: ……………………………………………………………………………………

**III. Employment**

1. How many people in total work in your initiative?

Male: Female:

1. How long do members/employees on average stay in the group?................................

1. How many work permanently or casually?

|  | Women | Men |
| --- | --- | --- |
| Permanent workers? |  |  |
| Temporary/casual employees? |  |  |

1. What is the job of the casual workers? Explain…………………………………………………………

………………………………………………………………………………………………………………………………….

1. What kind of training have the members of the initiative received?

...............................................................................................................................................

1. What is the average age and number of the members/employees? (Consider table below)

| Age (years) | Number |
| --- | --- |
| Under 16 |  |
| 16 to 25 |  |
| 26 to 35 |  |
| 36 to 49 |  |
| 49 to 60 |  |
| Over 60 |  |

1. What is the labor division by gender? Specify by number of men and women

|  | Women | Men |
| --- | --- | --- |
| collection |  |  |
| separation |  |  |
| processing |  |  |
| commercialization |  |  |
| administration |  |  |
| clean-ups |  |  |
| other activities |  |  |

**IV. Relationships**

1. Do you maintain relations with other waste picker groups?
   - - Yes No:
     - Name the groups
     - Nature of the relation:
2. Do you belong to a waste picker network?
   - - Yes: No:
     - Name of the network:
     - Purpose of the network:
     - Your role in the network:
