# Supplementary material for: Characteristics, challenges and innovations of waste picker organizations: A comparative perspective between Latin American and East African countries
Source: PLoS One. 2022 Jul 29;17(7):e0265889. doi: 10.1371/journal.pone.0265889 (PMC9337677; doi:10.1371/journal.pone.0265889)
Supplement: S2 Appendix — (DOCX) [file pone.0265889.s002.docx]

***Recycling networks***

***Mapping waste governance***

**Interview Guide**

**Name of interviewer:**

**Name of respondent:**

**Contact information of respondent:**

**Position within the initiative:**

**Date:**

**(Taking notes and recording)**

1. How was the initiative started? Please, share with us the origin of this initiative (When? How? With whom? Where? Why?)
2. What difficulties did you experience since your initiative started, and how did you address them? (e.g. Commercialization; management; financial sustainability; social inclusion (e.g. social security, human rights, children labour, gender inclusiveness…); legal challenges; relationships with other stakeholders (government, community, etc.).
3. What opportunities did the initiative have in the past? What did you do to take advantage of these opportunities?
4. What are the key achievements since you started the initiative?
5. What are the innovations that your initiative has developed? Explain how particular innovations were articulated (what challenges addressed, actors involved, changes and effects, resources mobilized, context of the innovation, etc).

- Recyclable materials?
- Technology innovations? (sorting, processing, transport)
- Networking and governance? (communities, companies, local governments, NGOs)
- Commercialization
- Management (internal)
- Social inclusion (e.g. social security, human rights, children labour, gender inclusiveness)

1. What opportunities do you envision in your initiative in the future?
